# Characteristics, Practice Patterns, and Outcomes of Emergency Department Intubation for Sepsis: An ANZEDAR Registry Study

**DOI:** 10.1111/1742-6723.70362

**Published:** 2026-10-08

**Authors:** Pramod Chandru, Hatem Alkhouri, Toby Fogg, Elliot Long

**Affiliations:** ^1^ Emergency Department Nepean Hospital Sydney New South Wales Australia; ^2^ Agency for Clinical Innovation Emergency Care Institute Sydney New South Wales Australia; ^3^ Faculty of Medicine The University of Sydney New South Wales Australia; ^4^ Emergency Department Royal North Shore Hospital Sydney New South Wales Australia; ^5^ CareFlight Sydney New South Wales Australia; ^6^ Royal Children's Hospital, Murdoch Children's Research Institute University of Melbourne Victoria Australia

## Abstract

**Objective:**

Sepsis is a leading cause of critical illness in Australia and New Zealand, with over 75% of admissions occurring through emergency departments. This study aimed to characterise the demographics, process of care, pharmacology, and peri‐intubation adverse events of sepsis intubations using a prospective Australasian registry.

**Methods:**

A retrospective analysis of prospectively collected data from the Australian and New Zealand Emergency Department Airway Registry (ANZEDAR) was performed across 79 hospitals between January 2010 and March 2026. Intubations performed for a primary indication of sepsis were identified. A paediatric subgroup analysis was performed stratified by age band. Statistical comparisons used Mann–Whitney *U* tests for continuous variables and chi‐square or Fisher's exact tests for categorical variables.

**Results:**

Of 11,289 registry intubations, 280 (2.5%) were performed for sepsis. Paediatric patients with confirmed age under 16 years comprised 30 of 280 sepsis intubations (10.7%). Adults were older, more haemodynamically compromised, and had longer triage to intubation times than non‐sepsis patients across all age groups. Laryngoscopic views and first‐pass success were comparable between sepsis and non‐sepsis groups. Haemodynamic complications occurred in 27.9% of sepsis intubations and peri‐intubation cardiac arrest in 4.6%. Waveform capnography was used less frequently in sepsis versus non‐sepsis groups (87.5% vs. 94.1%; *p* < 0.001).

**Conclusion:**

Emergency department sepsis intubation is associated with high rates of haemodynamic complications despite similar laryngoscopic views and first‐pass success rates. Low waveform capnography use in infant sepsis represents a potential patient safety concern. These findings support targeted interventions.

## 1 Introduction

Sepsis is a major cause of disease burden internationally, with over 48 million cases and 11 million sepsis‐related deaths estimated annually [1]. In Australia, sepsis accounted for 84,382 public hospital admissions in 2022–2023, with a 14.5% in‐hospital mortality rate [2]. These patients tend to be older, predominantly male, and highly comorbid. Recent data demonstrate 77.9% of sepsis admissions occur through emergency departments (ED) [2].

Although intubation may be required in sepsis for refractory respiratory failure, haemodynamic instability, or deteriorating conscious state, it is not included in the Surviving Sepsis Campaign's early management bundle, which prioritises antibiotic administration, fluid resuscitation and vasopressor initiation [3, 4]. The relationship between intubation timing and outcomes in sepsis is contested: in one retrospective cohort of patients with septic shock, earlier intubation was associated with lower 30‐day mortality, with another demonstrating ICU‐level variation in intubation frequency that has been associated with paradoxically higher mortality at centres with the highest intubation rates, suggesting optimal timing remains uncertain [5, 6].

Patients with sepsis frequently present with significant physiological derangement—including haemodynamic instability, reduced physiological reserve, and metabolic compromise—each of which is associated with higher rates of peri‐intubation adverse events in critically ill patients more broadly [7].

Paediatric sepsis patients represent a particularly high‐risk subgroup, with age‐specific physiology and haemodynamic vulnerability distinct from adult presentations [8]. The pharmacological and equipment requirements for paediatric emergency intubation differ substantially from adult practice, and specialist involvement is frequently required [9].

Previous analyses of the ANZEDAR registry have described overall intubation practice and outcomes across all indications, and characterised the paediatric emergency airway population [9, 10]. However, no registry‐based study has specifically examined the sepsis intubation cohort in the ED setting, describing the indication‐specific demographics, process of care adaptations, pharmacological decision‐making, and peri‐intubation complications that distinguish this physiologically high‐risk group from other intubation indications. This study aimed to characterise the demographics, process of care, pharmacology, first‐pass success, and peri‐intubation adverse events of ED sepsis intubation using the Australian and New Zealand Emergency Department Airway Registry (ANZEDAR), and to compare these with non‐sepsis ED intubations. A secondary aim was to describe practice patterns and outcomes within a paediatric sepsis subgroup.

## 2 Methods

A retrospective analysis of prospectively collected data was performed from participating sites (*n* = 79) across Australia and New Zealand as part of the ANZEDAR study, covering intubations recorded between January 2010 and March 2026.

After each intubation, outcomes were self‐reported by the intubating doctor or team leader using the ANZEDAR data collection form, with the Principal Investigator at each ED collating and checking data for completeness against medical records. Sites required at least 90% of intubation episodes captured over a minimum of 6 months for eligibility.

The unit of analysis was the intubation episode; patients with multiple episodes may contribute more than one record. Variable definitions have been described previously [9, 10]. In summary, an intubation attempt was defined as a single laryngoscope pass; an episode encompassed all attempts. Sepsis was identified by treating clinician coding of the primary indication as ‘Medical—Sepsis’ and was not validated against Sepsis‐3 criteria. Intubator experience, laryngoscope type, Cormack‐Lehane view, adjunct device use, preoxygenation, medications, complications and disposition were recorded. Peri‐intubation complications were defined as adverse events occurring during or immediately following the intubation episode. Complication definitions including graded desaturation thresholds and age‐appropriate haemodynamic parameters have been described previously [9, 10].

### 2.1 Statistical Analysis

Continuous variables are reported as median and interquartile range (IQR); categorical variables as frequency and percentage. Key between‐group differences are reported with 95% confidence intervals. All percentages use valid *N* as denominator; missing data are reported and were not imputed. No formal sample size calculation was performed.

Between‐group comparisons used Mann–Whitney *U* tests for continuous variables and chi‐square or Fisher's exact tests for categorical variables. Given the exploratory nature of this analysis, no adjustment for multiple comparisons was made; some associations may represent chance findings requiring prospective confirmation.

Binary logistic regression identified independent predictors of peri‐intubation haemodynamic complications within the sepsis cohort. Triage SBP was selected a priori as the candidate predictor, entered as a continuous variable. Complete case analysis was used (*n* = 245 of 280). Model fit was assessed using McFadden *R* ^2^. Results are reported as odds ratios with 95% CIs.

A paediatric subgroup analysis was stratified into three age bands: 0–< 2, 2–< 5 and 5–16 years. Given small sample sizes, all paediatric estimates should be considered hypothesis‐generating only. Key process of care comparisons with age‐matched registry intubations are provided in Table S1. Statistical significance was defined as *p* < 0.05. All analyses used Jamovi version 2.6 [11].

#### 2.1.1 Ethics

The Australia and New Zealand Emergency Department Airway Registry (ANZEDAR) project was approved by the Northern Sydney Local Health District Human Research Ethics Committee in 2012 (ID: 1209‐318M) and registered with the Australia New Zealand Trials Registry ACTRN12613001052729.

## 3 Results

### 3.1 Study Cohort

During the study period, 11,289 ED intubations were recorded across the registry. Data were drawn from 79 hospitals across Australia and New Zealand: 29 major referral, 22 urban district, 25 rural/regional (including one remote centre), and three paediatric‐only centres. Of these, 280 (2.5%) were performed for a primary indication of sepsis, forming the study cohort (Figure 1); the remaining 11,009 served as the comparator group. Sepsis was the ninth most common intubation indication but accounted for 6.7% of all intubations in patients aged under 2 years.

**FIGURE 1:**
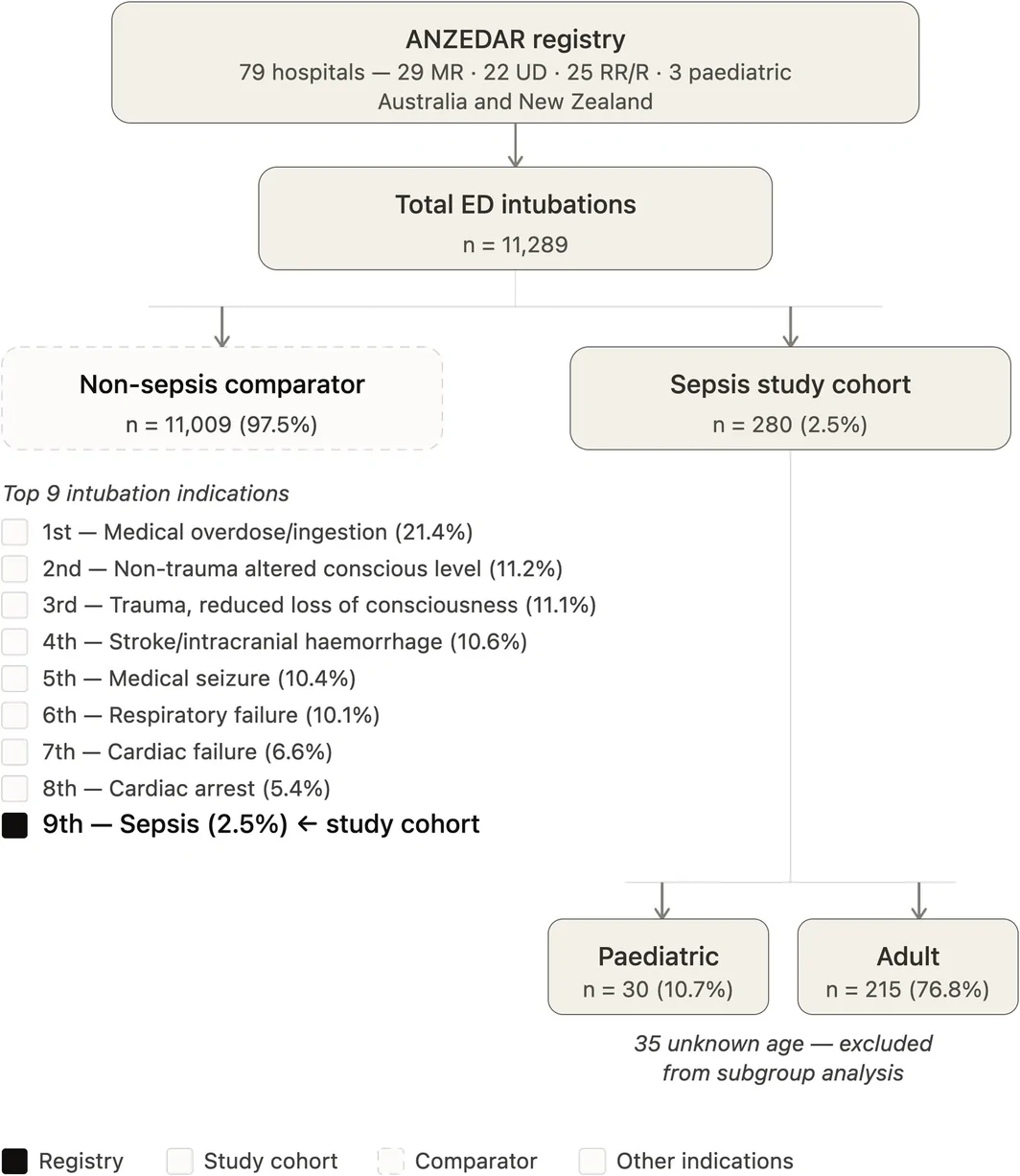
Cohort selection from the Australian and New Zealand Emergency Department Airway Registry (ANZEDAR). MR, major referral; RR/R, rural/regional; UD, urban district.

### 3.2 Sepsis Cohort Characteristics

Sepsis patients were older than the general registry population (median age 60 years, IQR 40–70 vs. 50 years, IQR 31–66; *p* < 0.001) and presented with greater haemodynamic instability, including lower triage systolic blood pressure (98 mmHg, IQR 86–118 vs. 130 mmHg, IQR 110–150), higher heart rate (120 vs. 100 bpm), higher respiratory rate (30 vs. 18 breaths/min), and lower SpO_2_ (98% vs. 99%; all *p* < 0.001), despite a higher triage GCS (12 vs. 6; *p* < 0.001). Triage to intubation time was significantly longer in sepsis (median 121.5 min, IQR 65–238 vs. 44 min, IQR 23–109; *p* < 0.001). Post‐intubation haemodynamic compromise persisted, with lower SBP (105 vs. 125 mmHg) and higher HR (120 vs. 103 bpm; both *p* < 0.001). Laryngoscopic view was comparable between groups (*p* = 0.543), with Grade I/II views in 85.6% and Grade III/IV in 14.4% of sepsis intubations (Table 1).

**TABLE 1:** Baseline characteristics and intubation parameters—sepsis versus non‐sepsis.

Variable	Non‐sepsis (*n* = 11,009) median (IQR) or *n*/*N* (%)	Sepsis (*n* = 280) median (IQR) or *n*/*N* (%)	*p*
Patient characteristics			
Age, years—median (IQR)	50 (31–66) *n* = 10,664 missing = 345	60 (40–70) *n* = 245 missing = 35	< 0.001
Weight, kg—median (IQR)	75 (65–90) *n* = 10,358 missing = 651	70 (50–85) *n* = 262 missing = 18	< 0.001
GCS—median (IQR)	6 (3–9) *n* = 10,617 missing = 392	12 (6–14) *n* = 245 missing = 35	< 0.001
Triage SBP, mmHg—median (IQR)	130 (110–150) *n* = 9760 missing = 1249	98 (86–118) *n* = 245 missing = 35	< 0.001
Triage HR, bpm—median (IQR)	100 (80–120) *n* = 9898 missing = 1111	120 (100–143) *n* = 253 missing = 27	< 0.001
Triage RR, breaths/min—median (IQR)	18 (14–24) *n* = 9042 missing = 1967	30 (22–40) *n* = 235 missing = 45	< 0.001
Triage SpO_2_, %—median (IQR)	99 (96–100) *n* = 9928 missing = 1081	98 (92–100) *n* = 240 missing = 40	< 0.001
Post‐intubation SBP, mmHg—median (IQR)	125 (105–150) *n* = 8746 missing = 2263	105 (85–129) *n* = 215 missing = 65	< 0.001
Triage to intubation, min—median (IQR)	44 (23–109) *n* = 5562 missing = 5447	121.5 (65–238) *n* = 138 missing = 142	< 0.001
Intubation parameters			
Cormack‐Lehane Grade I/II	8171/9645 (84.7%) missing = 1364	208/243 (85.6%) missing = 37	0.543
Cormack‐Lehane Grade III/IV	1474/9645 (15.3%) missing = 1364	35/243 (14.4%) missing = 37	0.543
Cuffed ETT	4782/4861 (98.4%) missing = 6148	115/119 (96.6%) missing = 161	0.272
External laryngeal manipulation	2020/10,330 (19.6%) missing = 679	42/260 (16.2%) missing = 20	0.198
Bougie use	7465/10,715 (69.7%) missing = 294	150/266 (56.4%) missing = 14	< 0.001
Stylet use	1631/10,715 (15.2%) missing = 294	57/266 (21.4%) missing = 14	0.007
Neither bougie nor stylet	1611/10,715 (15.0%) missing = 294	59/266 (22.2%) missing = 14	0.002
Cricoid pressure	2046/10,673 (19.2%) missing = 336	32/268 (11.9%) missing = 12	0.004
Manual in‐line stabilisation	1518/10,709 (14.2%) missing = 300	3/266 (1.1%) missing = 14	< 0.001
Age distribution			
Paediatric (< 16 years)	720/10,664 (6.8%)	30/245 (12.2%)	< 0.001
0–< 2 years	83/10,664 (0.8%)	6/245 (2.4%)	
2–< 5 years	168/10,664 (1.6%)	10/245 (4.1%)	
5–16 years	469/10,664 (4.4%)	14/245 (5.7%)	
Adult (≥ 16 years)	9944/10,664 (93.2%)	215/245 (87.8%)	
Age missing	345 (3.1%)	35 (12.5%)	
Intubation attempts			
Single attempt	9618/11,009 (87.4%)	240/280 (85.7%)	0.462
Two attempts	1131/11,009 (10.3%)	29/280 (10.4%)	
Three or more attempts	260/11,009 (2.4%)	11/280 (3.9%)	

*Note:* All percentages use valid *N* as denominator. Missing data reported for all variables with missing > 0.

Abbreviations: ETT, endotracheal tube; GCS, Glasgow Coma Scale; HR, heart rate; IQR, interquartile range; RR, respiratory rate; SBP, systolic blood pressure.

### 3.3 Process of Care and Pharmacology

Sepsis patients were more likely to be intubated by a specialist (31% vs. 21%; difference +10.0%, 95% CI: 4.5–15.5; *p* = 0.001) with greater ICU involvement (10% vs. 4%; *p* < 0.001). Video laryngoscopy was used less frequently in sepsis (60% vs. 70%; difference −9.5%, 95% CI: −15.4 to −3.7; *p* < 0.001) and waveform capnography confirmation was lower (87.5% vs. 94.1%; difference −6.7%, 95% CI: −10.8 to −2.6; *p* < 0.001), with clinical confirmation alone in 5.5% versus 2.1% of cases (*p* = 0.001). Checklist use and first‐pass success were similar between groups (Table 2).

**TABLE 2:** Process of care and pharmacology—sepsis versus non‐sepsis.

Variable	Non‐sepsis (*n* = 11,009) *n*/*N* (%)	Sepsis (*n* = 280) *n*/*N* (%)	*p*
Operator			
Specialist intubator	2320/10,914 (21.3%) missing = 95	87/278 (31.3%) missing = 2	0.001
ICU intubator	472/10,941 (4.3%) missing = 68	28/278 (10.1%) missing = 2	< 0.001
Predicted difficult airway	3400/10,979 (31.0%) missing = 30	76/279 (27.2%) missing = 1	0.206
Device			
Video laryngoscopy (any)	7584/10,882 (69.7%) missing = 127	166/276 (60.1%) missing = 4	< 0.001
CMAC	6520/10,882 (59.9%)	141/276 (51.1%)	
MacIntosh	2500/10,882 (23.0%)	75/276 (27.2%)	
Miller	96/10,882 (0.9%)	15/276 (5.4%)	
Glidescope	613/10,882 (5.6%)	12/276 (4.3%)	
Other VL	430/10,882 (4.0%)	12/276 (4.3%)	
Process			
Pre‐RSI checklist used	6736/9629 (70.0%) missing = 1380	175/243 (72.0%) missing = 37	0.534
Waveform capnography	9350/9932 (94.1%) missing = 1077	223/255 (87.5%) missing = 25	< 0.001
Colour change capnography	378/9932 (3.8%) missing = 1077	18/255 (7.1%) missing = 25	0.013
Clinical confirmation alone	204/9932 (2.1%) missing = 1077	14/255 (5.5%) missing = 25	0.001
First pass success	9533/10,977 (86.8%) missing = 32	239/280 (85.4%) missing = 0	0.524
Pharmacology			
Ketamine	4360/11,009 (39.6%)	176/280 (62.9%)	< 0.001
Dose mg—median (IQR)	100 (50–100) *n* = 4360	80 (45–100) *n* = 176	0.003
Propofol	3333/11,009 (30.3%)	36/280 (12.9%)	< 0.001
Dose mg—median (IQR)	80 (50–100) *n* = 3333	55 (40–100) *n* = 36	0.022
Thiopentone	1862/11,009 (16.9%)	25/280 (8.9%)	0.001
Rocuronium	5724/11,009 (52.0%)	168/280 (60.0%)	0.010
Dose mg—median (IQR)	100 (100–150) *n* = 5724	100 (50–100) *n* = 168	< 0.001
Suxamethonium	4274/11,009 (38.8%)	98/280 (35.0%)	0.217
Dose mg—median (IQR)	100 (100–150) *n* = 4274	100 (100–150) *n* = 98	0.101
Fentanyl	2869/11,009 (26.1%)	69/280 (24.6%)	0.642
Dose mcg—median (IQR)	100 (50–100) *n* = 2869	75 (20–100) *n* = 69	0.001
Etomidate	101/11,009 (0.9%)	3/280 (1.1%)	1.000
Midazolam	642/11,009 (5.8%)	14/280 (5.0%)	0.647
Any vasopressor (free‐text)	156/11,009 (1.4%)	15/280 (5.4%)	< 0.001

*Note:* Drug doses reported for recipients only (dose > 0). Vasopressor use captured via free‐text field only—likely underestimates true use. Predicted difficult airway *p* = 0.206—not significant (corrected from previous version).

Abbreviations: EM, emergency medicine; ICU, intensive care unit; RSI, rapid sequence induction; VL, video laryngoscopy.

Ketamine was used more frequently (63% vs. 40%; difference +23.3%, 95% CI: 17.5–29.0; *p* < 0.001) and at lower median doses (80 vs. 100 mg; *p* = 0.003). Propofol and thiopentone were used less frequently (both *p* < 0.001) and at lower doses. Rocuronium use was higher (60% vs. 52%; *p* = 0.010). Fentanyl doses were lower in sepsis recipients (75 vs. 100 mcg; *p* = 0.001). Vasopressor use was higher in sepsis (5% vs. 1%; *p* < 0.001). Etomidate use was low in both groups (1%) (Table 2).

### 3.4 Complications and Outcomes

Any desaturation occurred in 22.5% of sepsis intubations versus 11.9% in non‐sepsis (difference +10.6%, 95% CI: 5.7–15.5; *p* < 0.001). Critical desaturation and any respiratory complication did not differ significantly between groups (Table 3).

**TABLE 3:** Peri‐intubation complications and outcomes—sepsis versus non‐sepsis.

Complication/outcome	Non‐sepsis (*n* = 11,009) *n*/*N* (%)	Sepsis (*n* = 280) *n*/*N* (%)	*p*
Respiratory			
Any desaturation	1308/11,009 (11.9%)	63/280 (22.5%)	< 0.001
Desaturation type 1	371/11,009 (3.4%)	20/280 (7.1%)	0.001
Desaturation type 2	81/11,009 (0.7%)	3/280 (1.1%)	0.769
Sustained desaturation (> 60s)	85/11,009 (0.8%)	3/280 (1.1%)	0.827
Critical desaturation (< 70%)	149/11,009 (1.4%)	5/280 (1.8%)	0.723
Any respiratory complication	682/11,009 (6.2%)	23/280 (8.2%)	0.170
Oesophageal intubation	198/11,009 (1.8%)	9/280 (3.2%)	0.129
Aspiration	143/11,009 (1.3%)	5/280 (1.8%)	0.659
Mainstem intubation	233/11,009 (2.1%)	6/280 (2.1%)	1.000
Surgical airway	35/11,009 (0.3%)	0/280 (0.0%)	0.689
Haemodynamic			
Any haemodynamic complication	1480/11,009 (13.4%)	78/280 (27.9%)	< 0.001
Hypotension—any definition	959/11,009 (8.7%)	57/280 (20.4%)	< 0.001
Adult hypotension	567/11,009 (5.2%)	23/280 (8.2%)	0.032
Paediatric hypotension	45/11,009 (0.4%)	8/280 (2.9%)	< 0.001
Unspecified hypotension	347/11,009 (3.2%)	26/280 (9.3%)	< 0.001
Cardiac arrest	163/11,009 (1.5%)	13/280 (4.6%)	< 0.001
Adult tachycardia	131/11,009 (1.2%)	6/280 (2.1%)	0.245
Paediatric tachycardia	14/11,009 (0.1%)	7/280 (2.5%)	< 0.001
Adult bradycardia	28/11,009 (0.3%)	1/280 (0.4%)	1.000
Hypertension	210/11,009 (1.9%)	1/280 (0.4%)	0.095
Outcome			
Disposition—ICU	7704/10,444 (73.8%) missing = 565	208/269 (77.3%) missing = 11	0.214
Disposition—Transfer	1000/10,444 (9.6%) missing = 565	29/269 (10.8%) missing = 11	0.577
Disposition—Theatre	943/10,444 (9.0%) missing = 565	13/269 (4.8%) missing = 11	0.023
Extubated in ED	213/10,444 (2.0%) missing = 565	0/269 (0.0%) missing = 11	0.032
Re‐intubation in ED	23/10,444 (0.2%) missing = 565	1/269 (0.4%) missing = 11	1.000
ED mortality	561/10,444 (5.4%) missing = 565	18/269 (6.7%) missing = 11	0.419

*Note:* Hypotension reported as combined adult, paediatric and unspecified definitions. Disposition denominator excludes missing (sepsis *n* = 269, non‐sepsis *n* = 10,444). ED mortality *p* = 0.419—corrected from previous version (table showed 0.0345; text showed 0.345; both incorrect). Overall disposition chi‐square *p* = 0.027 reflects all six disposition categories combined. Adult tachycardia *p* = 0.245—not significant.

Any haemodynamic complication occurred in 27.9% of sepsis intubations versus 13.4% in non‐sepsis (difference +14.4%, 95% CI: 9.1–19.7; *p* < 0.001). Combined hypotension affected 20.4% versus 8.7% (*p* < 0.001). Peri‐intubation cardiac arrest occurred in 4.6% versus 1.5% (difference +3.2%, 95% CI: 0.7–5.6; *p* < 0.001)—a three‐fold higher rate. Adult tachycardia, hypertension, and bradycardia did not differ significantly. No surgical airways were performed in the sepsis cohort.

Sepsis patients were less likely to be transported to theatre (4.8% vs. 9.0%; *p* = 0.023) and none were extubated in the ED (0% vs. 2.0%; *p* = 0.032). ED mortality and ICU admission did not differ significantly between groups (Table 3).

Binary logistic regression (*n* = 245) identified lower triage SBP as the only independent predictor of haemodynamic complications (OR 0.98 per mmHg, 95% CI: 0.97–0.99; *p* = 0.003), with each 10 mmHg reduction associated with approximately 20% higher odds.

### 3.5 Paediatric Subgroup

Thirty sepsis patients had a confirmed age under 16 years (10.7% of the cohort), disproportionate to their 6.8% prevalence in the overall registry. Sepsis accounted for 6.7% of all intubations in patients under 2 years compared with 2.1% of adult intubations. Physiological derangement was greatest in the youngest age groups, with median triage SBP of 60.5 mmHg and HR of 176 bpm in infants. Vasopressor use was significantly higher in younger age groups (*p* = 0.003), with 33.3% in infants compared with 4.2% in adults. Triage to intubation time was longer in sepsis across all age groups compared with non‐sepsis comparators (Table 4).

**TABLE 4:** Paediatric sepsis subgroup—key characteristics by age band.

Variable	0– < 2 years (*n* = 6)	2– < 5 years (*n* = 10)	5–16 years (*n* = 14)^b^	Adult (*n* = 215)	*p* ^a^
Physiology—median (IQR)					
Triage SBP, mmHg	60.5 (60–72)	81 (64–100)	108 (99–117)	100 (90–123)	0.002
Triage HR, bpm	176 (166–189)	170 (165–194)	140 (128–151)	110 (93–130)	< 0.001
Triage RR, breaths/min	50 (40–63)	44 (40–48)	38 (27–46)	28 (20–36)	< 0.001
Triage to intubation, min (missing: 0, 3, 2, 120)	139 (94–474)	70 (60–99)	55 (46–99)	150 (76–272)	0.029
Operator—*n*/*N* (%)					
EM intubator	1/6 (16.7%)	1/10 (10.0%)	3/14 (21.4%)	187/214 (87.4%)	< 0.001^c^
ICU intubator	2/6 (33.3%)	2/10 (20.0%)	5/14 (35.7%)	11/214 (5.1%)	< 0.001^c^
Paediatric specialty^d^	0/6 (0.0%)	1/10 (10.0%)	1/14 (7.1%)	0/214 (0.0%)	< 0.001^c^
Specialist team leader	4/6 (66.7%)	4/10 (40.0%)	8/14 (57.1%)	58/214 (27.1%)	0.019^c^
Device—*n*/*N* (%)					
Video laryngoscopy (any)	2/6 (33.3%)	5/10 (50.0%)	11/14 (78.6%)	133/212 (62.7%)	0.223^c^
Miller blade	1/6 (16.7%)	0/10 (0.0%)	1/14 (7.1%)	0/212 (0.0%)	< 0.001^c^
Bougie use	0/5 (0.0%)	1/10 (10.0%)	3/12 (25.0%)	144/207 (69.6%)	< 0.001^c^
Stylet use	3/5 (60.0%)	2/10 (20.0%)	4/12 (33.3%)	31/207 (15.0%)	0.024^c^
Process—*n*/*N* (%)					
Waveform capnography	3/6 (50.0%)	9/10 (90.0%)	11/13 (84.6%)	177/196 (90.3%)	0.020^c^
Clinical confirmation alone	2/6 (33.3%)	1/10 (10.0%)	2/13 (15.4%)	5/196 (2.6%)	< 0.001^c^
First pass success	6/6 (100.0%)	9/10 (90.0%)	10/14 (71.4%)	187/215 (87.0%)	0.282^c^
Checklist used	3/6 (50.0%)	8/10 (80.0%)	9/13 (69.2%)	133/186 (71.5%)	0.632^c^
Pharmacology—*n*/*N* (%)					
Ketamine	6/6 (100.0%)	7/10 (70.0%)	11/14 (78.6%)	137/215 (63.7%)	0.202^c^
Rocuronium	6/6 (100.0%)	9/10 (90.0%)	13/14 (92.9%)	121/215 (56.3%)	0.002^c^
Suxamethonium	0/6 (0.0%)	2/10 (20.0%)	1/14 (7.1%)	83/215 (38.6%)	0.017^c^
Fentanyl	0/6 (0.0%)	1/10 (10.0%)	4/14 (28.6%)	46/215 (21.4%)	0.417^c^
Any vasopressor	2/6 (33.3%)	2/10 (20.0%)	2/14 (14.3%)	9/215 (4.2%)	0.003^c^
Complications—*n*/*N* (%)					
Any haemodynamic complication	1/6 (16.7%)	4/10 (40.0%)	3/14 (21.4%)	60/215 (27.9%)	0.706^c^
Adult hypotension	0/6 (0.0%)	0/10 (0.0%)	0/14 (0.0%)	23/215 (10.7%)	0.315^c^
Paediatric hypotension	0/6 (0.0%)	3/10 (30.0%)	2/14 (14.3%)	0/215 (0.0%)	< 0.001^c^
Paediatric tachycardia	1/6 (16.7%)	0/10 (0.0%)	0/14 (0.0%)	0/215 (0.0%)	< 0.001^c^
Cardiac arrest	0/6 (0.0%)	2/10 (20.0%)	1/14 (7.1%)	10/215 (4.7%)	0.178^c^
Any desaturation	1/6 (16.7%)	1/10 (10.0%)	5/14 (35.7%)	50/215 (23.3%)	0.504^c^
Disposition—*n*/*N* (%)					
ICU admission	4/6 (66.7%)	3/10 (30.0%)	10/14 (71.4%)	169/205 (82.4%)	< 0.001^c^
Transfer to another hospital	2/6 (33.3%)	5/10 (50.0%)	4/14 (28.6%)	10/205 (4.9%)	< 0.001^c^
Died in ED	0/6 (0.0%)	1/10 (10.0%)	0/14 (0.0%)	15/205 (7.3%)	0.636^c^

Abbreviations: EM, emergency medicine; HR, heart rate; ICU, intensive care unit; RR, respiratory rate; SBP, systolic blood pressure.

^a^ *p* value compares across all four sepsis age groups.

^b^ 5–16 years (*n* = 14) and 2– < 5 years (*n* = 10): interpret with extreme caution. 0– < 2 years (*n* = 6): all estimates exploratory only.

^c^ Small expected cell counts in paediatric age groups—chi‐square test assumptions may not be met. All *p* values should be interpreted with caution.

^d^ Paediatric specialty includes paediatric emergency medicine, paediatrics, and neonatal/paediatric retrieval (NETS).

The intubating specialty differed significantly by age group (*p* < 0.001), with a sepsis‐specific escalation pattern absent in adults. Emergency medicine intubated 16.7% of infant sepsis cases compared with 37.3% of non‐sepsis infants, falling further in 2–< 5 years (10.0% vs. 49.1%) and 5–16 years (21.4% vs. 55.8%) cases, with corresponding increases in ICU and paediatric specialty involvement. Adult rates were similar regardless of indication (87.4% vs. 87.9%) (Table S1).

Waveform capnography use was lower in infant sepsis (3/6, 50.0%) compared with non‐sepsis infants (79/83, 95.2%); however, this estimate is based on only six infant sepsis intubations and should be interpreted with extreme caution. Clinical confirmation alone was used in 2/6 (33.3%) infant sepsis cases versus 4/83 (4.8%) of non‐sepsis infants.

First pass success was comparable across all age groups (*p* = 0.282). Inter‐hospital transfer occurred in 28%–50% of paediatric sepsis cases compared with 4.9% of adults. The 2–< 5 years group had the highest rates of cardiac arrest (20.0%) and hypotension (30.0%), though all estimates must be interpreted with extreme caution given *n* = 10 (Table 4).

## 4 Discussion

In this analysis of the ANZEDAR registry we identified a clinically and physiologically distinct sepsis cohort with high rates of peri‐intubation complications. Adult patients were older and more haemodynamically compromised, with prolonged triage to intubation time consistent with either intentional pre‐intubation resuscitation or delayed recognition of the need for airway intervention—a distinction with significant implications that our data cannot resolve. Paediatric patients (confirmed age < 16 years) comprised 10.7% of the sepsis cohort, disproportionate to their 6.8% registry prevalence, with a sepsis‐specific multi‐specialty escalation pattern and age‐appropriate device and drug selection. Despite comparable laryngoscopic views and first‐pass success rates, haemodynamic complications occurred in 27.9% of sepsis intubations and peri‐intubation cardiac arrest in 4.6%—a three‐fold higher rate than non‐sepsis intubations—consistent with a physiological rather than anatomical challenge. A potential patient safety concern was identified in lower waveform capnography use in sepsis overall (87.5% vs. 94.1%). Waveform capnography use in infant sepsis was 3/6 (50%) compared with 79/83 (95.2%) in non‐sepsis infants; however this estimate is based on only six cases and should be interpreted with caution.

The timing of intubation in sepsis remains an area of ongoing research with conflicting results [5, 6]. In our cohort, triage to intubation time was significantly longer in sepsis than non‐sepsis across all age groups—0–< 2 years: 139 versus 48 min, 2–< 5 years: 70 versus 47 min, 5–16 years: 55 versus 49 min, adults: 150 versus 42 min—a consistent sepsis‐associated delay across all age groups that was not explained by age or indication. Two interpretations are plausible: the delay may reflect intentional haemodynamic resuscitation prior to intubation—a clinically appropriate strategy aligned with current sepsis guidelines—or delayed recognition of the need for airway intervention in patients presenting with primarily physiological rather than overt airway compromise. Our registry data cannot distinguish between these interpretations, and the distinction has significant clinical implications given emerging evidence that intubation timing may influence outcomes in septic shock [5]. In paediatric sepsis specifically, earlier intubation may reduce oxygen demand and improve delivery to critical organs, though current guidelines do not specify whether to intubate children with sepsis in the absence of respiratory failure [4, 8].

Sepsis was associated with a measurable specialist escalation response in paediatric intubations, largely absent in adults. Emergency medicine intubator rates were similar in adults (87.4% vs. 87.9%) but fell substantially in paediatric sepsis—0–< 2 years: 16.7% versus 37.3%, 2–< 5 years: 10.0% versus 49.1%, 5–16 years: 21.4% versus 55.8%—with corresponding increases in ICU and paediatric specialty involvement. This suggests either that paediatric sepsis is recognised as a distinct high‐risk scenario or that its physiological severity drives a compensatory staffing response. Poor teamwork has been independently associated with hypoxaemia and adverse events in shock‐indicated paediatric intubations, supporting multidisciplinary simulation training for this population [12]. This specialist‐dependent model has implications for regional and after‐hours settings where such expertise may be unavailable, potentially explaining the high inter‐hospital transfer rates in paediatric sepsis (28%–50% across age groups vs. 4.9% in adults).

Our data are consistent with the primary challenge in sepsis intubation being physiological rather than anatomical. Despite comparable laryngoscopic views and first‐pass success rates, sepsis patients experienced a three‐fold higher peri‐intubation cardiac arrest rate (4.6% vs. 1.5%) and haemodynamic complications in 27.9% of cases. The low rate of etomidate use (1%) is consistent with its lack of Therapeutic Goods Administration approval in Australia and clinician avoidance of adrenal suppression concerns, though its evidence base as a haemodynamically favourable alternative to ketamine in sepsis remains under active investigation [13, 14, 15].

Waveform capnography use was lower in sepsis overall (87.5% vs. 94.1%), with a notably lower rate observed in infant sepsis (3/6, 50%) compared with non‐sepsis infants (79/83, 95.2%), though this should be interpreted cautiously given the very small infant sepsis sample [16, 17]. Two mechanisms likely contribute: technical limitations with small uncuffed tubes and limited equipment at non‐specialist sites; and reduced end‐tidal CO_2_ in low cardiac output states producing an absent waveform potentially misattributed to equipment failure rather than tube misplacement [18, 19]. These findings have implications for equipment procurement, clinician training, and system design—including retrieval team attendance prior to intubation or earlier transfer before deterioration necessitates intubation at non‐specialist sites.

## 5 Limitations

This study has several limitations. ANZEDAR captures data from voluntary sites, introducing potential selection bias toward departments with greater interest in airway quality improvement. Missing data across key variables—including triage to intubation time (missing in 50.7% of the sepsis cohort)—limits some analyses. Sepsis diagnosis relied on clinician coding rather than validated Sepsis‐3 criteria, with misclassification possible in both directions. Vasopressor use was captured via free‐text only, and the 5% rate likely underestimates true use. Pre‐hospital interventions are not captured. The registry cannot determine whether delayed intubation reflected intentional resuscitation or delayed recognition. Given the large number of comparisons performed, no adjustment for multiple comparisons was made; some findings may represent chance and require prospective confirmation. Intubations were clustered within 79 hospitals; clustering was not accounted for in the primary analysis, which may underestimate standard errors. Finally, the paediatric subgroup is substantially limited by small sample sizes, particularly in the 0–< 2 years (*n* = 6) and 2–< 5 years (*n* = 10) age bands; all paediatric estimates should be considered hypothesis‐generating only.

## 6 Conclusion

This registry‐based study provides the first characterisation of ED sepsis intubation practice in Australasia. Sepsis patients are physiologically distinct and high‐risk—older, more haemodynamically compromised, and intubated later—yet the primary challenge is haemodynamic rather than anatomical, with comparable laryngoscopic views and first‐pass success contrasting with a three‐fold higher peri‐intubation cardiac arrest rate and haemodynamic complications in 27.9% of cases. Waveform capnography confirmation was suboptimal in sepsis overall, representing a potential patient safety concern. Paediatric patients comprised 22% of the sepsis cohort, with a sepsis‐specific specialist escalation response absent in adults and inter‐hospital transfer rates of 28%–50% across paediatric age groups, consistent with intubation occurring at non‐specialist sites in a substantial proportion of cases. The convergence of suboptimal tube confirmation, specialist dependency, and high‐risk retrieval in the most vulnerable patients defines a potentially modifiable risk pathway warranting prospective evaluation and targeted quality improvement.

## Author Contributions

P.C., E.L. and H.A. drafted the manuscript. H.A. is the ANZEDAR coordinator. P.C. conceived the study and provided data and statistical analysis. All authors revised the manuscript and approved the final draft. T.F. is Principal Investigator of the ANZEDAR.

## Funding

This work was supported by the Emergency Care Institute.

## Ethics Statement

The ANZEDAR project was approved by the Northern Sydney Local Health District Human Research Ethics Committee in 2012 (ID: 1209‐318M).

## Conflicts of Interest

The authors declare no conflicts of interest.

## Supporting information


**Table S1:** Key process of care variables—sepsis versus all indications by age group.

## Data Availability

Data are available from the corresponding author on reasonable request. Public sharing is not permitted due to privacy/ethical restrictions.
