# Supplementary material for: Characteristics, Practice Patterns, and Outcomes of Emergency Department Intubation for Sepsis: An ANZEDAR Registry Study
Source: Emerg Med Australas. 2026 Oct 8;38(5):e70362. doi: 10.1111/1742-6723.70362 (PMC13647193; doi:10.1111/1742-6723.70362)
Supplement: Supplementary file 1 [file EMM-38-0-s001.docx]

**Supplementary Table 1 - Key Process of Care Variables — Sepsis vs All Indications by Age Group**

*Blue columns = sepsis patients. Gold columns = all intubations of same age regardless of indication (non-sepsis + sepsis combined), shown for contextual comparison.*

*‡Paediatric specialty includes paediatric EM, paediatrics, and NETS. §p-value for selected variables not calculated given small sepsis subgroup sizes — contextual comparison only.*

*Waveform capnography denominators: those with confirmation data recorded. Non-sepsis n for capnography: 0-<2yr=83, 2-<5yr=157, 5-16yr=438, adult=8,961.*

| **Variable** | **0-<2yr** | | **2-<5yr** | | **5-16yr** | | **Adult** | | **p-value§** |
| --- | --- | --- | --- | --- | --- | --- | --- | --- | --- |
|  | **Sepsis (n=6)** | **All (n=89)** | **Sepsis (n=10)** | **All (n=178)** | **Sepsis (n=14)** | **All (n=483)** | **Sepsis (n=215)** | **All (n=10,159)** |  |
| **Operator — n/N (%)** | | | | | | | | | |
| EM intubator | 1/6 (16.7%) | 31/83 (37.3%) | 1/10 (10.0%) | 82/167 (49.1%) | 3/14 (21.4%) | 260/466 (55.8%) | 187/214 (87.4%) | 8,701/9,896 (87.9%) |  |
| ICU intubator | 2/6 (33.3%) | 8/83 (9.6%) | 2/10 (20.0%) | 25/167 (15.0%) | 5/14 (35.7%) | 31/466 (6.7%) | 11/214 (5.1%) | 358/9,896 (3.6%) |  |
| Paediatric specialty‡ | 0/6 (0.0%) | 4/83 (4.8%) | 1/10 (10.0%) | 3/167 (1.8%) | 1/14 (7.1%) | 10/466 (2.1%) | 0/214 (0.0%) | 13/9,896 (0.1%) |  |
| **Process — n/N (%)** | | | | | | | | | |
| Waveform capnography | 3/6 (50.0%) | 79/83 (95.2%) | 9/10 (90.0%) | 148/157 (94.3%) | 11/13 (84.6%) | 406/438 (92.7%) | 177/196 (90.3%) | 8,456/8,961 (94.4%) |  |
| Clinical confirmation alone | 2/6 (33.3%) | 4/83 (4.8%) | 1/10 (10.0%) | 5/157 (3.2%) | 2/13 (15.4%) | 18/438 (4.1%) | 5/196 (2.6%) | 166/8,961 (1.9%) |  |
| First pass success | 6/6 (100.0%) | 68/83 (81.9%) | 9/10 (90.0%) | 143/167 (85.6%) | 10/14 (71.4%) | 418/466 (89.7%) | 187/215 (87.0%) | 8,651/9,929 (87.1%) |  |
| Video laryngoscopy (any) | 2/6 (33.3%) | 63/83 (75.9%) | 5/10 (50.0%) | 101/165 (61.2%) | 11/14 (78.6%) | 358/465 (77.0%) | 133/212 (62.7%) | 6,901/9,841 (70.1%) |  |
| **Disposition — n/N (%)** | | | | | | | | | |
| Transfer to another hospital | 2/6 (33.3%) | 17/83 (20.5%) | 5/10 (50.0%) | 28/162 (17.3%) | 4/14 (28.6%) | 94/453 (20.8%) | 10/205 (4.9%) | 795/9,433 (8.4%) |  |
| ICU admission | 4/6 (66.7%) | 61/83 (73.5%) | 3/10 (30.0%) | 124/162 (76.5%) | 10/14 (71.4%) | 307/453 (67.8%) | 169/205 (82.4%) | 7,010/9,433 (74.3%) |  |
